# The impact of smoking on patient outcomes in severe sepsis and septic shock

**DOI:** 10.1186/s40560-018-0312-x

**Published:** 2018-07-28

**Authors:** Fahad Alroumi, Ahmed Abdul Azim, Rachel Kergo, Yuxiu Lei, James Dargin

**Affiliations:** 1 Department of Pulmonary and Critical Care Medicine, Lahey Hospital and Medical Center, Burlington, MA USA; 2 Tufts University School of Medicine, Boston, MA USA

**Keywords:** Sepsis, Smoking, Septic shock, Mortality, Cigarettes, Tobacco

## Abstract

**Background:**

To assess, in the setting of severe sepsis and septic shock, whether current smokers have worse outcomes compared to non-smokers.

**Methods:**

This is a retrospective analysis of immunocompetent adult patients with severe sepsis and septic shock at a tertiary medical center. The primary outcome was the effect of active smoking on hospital mortality. Chi-square test and logistic regression were used to assess categorical outcomes. Wilcoxon rank-sum was utilized to test the differences in continuous outcomes among the varied smoking histories. Multivariable logistic regression was used to evaluate the association of smoking and mortality, need for vasopressors, mechanical ventilation, and ICU admission.

**Results:**

Of the 1437 charts reviewed, 562 patients were included. Current smokers accounted for 19% (107/562) of patients, while 81% (455/562) were non-smokers. The median hospital length of stay in survivors was significantly longer in current smokers versus non-smokers (8 vs 7 days, *p* = 0.03). There was a trend towards a higher mortality among current smokers, but this failed to meet statistical significance (OR 1.81, 95% CI 0.92–3.54, *p* = 0.08). On multivariable analysis, current smoking was associated with the need for mechanical ventilation (OR 2.38, 95% CI 1.06–5.34, *p* = 0.04), but that association was not observed with the need for vasopressors (OR 2.10, 95% CI 1.01–4.36, *p* = 0.58) nor ICU admission (OR 0.93, 95% CI 0.41–2.13, *p* = 0.86).

**Conclusions:**

In patients with severe sepsis or septic shock, current smoking was associated with a longer hospital stay, the need for mechanical ventilation, and trended towards a higher mortality. Larger multicenter prospective case-control studies are needed to confirm these findings.

## Background

Tobacco smoking remains the leading cause of preventable illness and death worldwide, accounting for approximately six million deaths annually [1]. Smoking imposes a heavy economic toll, costing countries billions of dollars in productivity and medical care [1]. In addition, it negatively impacts patient outcomes from acute illnesses, including influenza, pneumococcal pneumonia, and acute respiratory distress syndrome [2–4]. Although tobacco smoking is a risk factor for pulmonary and extra-pulmonary infections alike, its role in sepsis remains unclear [4–7].

Sepsis affects over one million individuals annually in the USA, and the mortality can be as high as 30% [8]. The costs incurred by sepsis have been estimated at $20 billion annually, making it one of the most expensive conditions treated in hospitals [8]. Smoking predisposes to infection through both structural and immunologic mechanisms [2]. Tobacco smoke causes peribronchiolar inflammation and fibrosis, which results in an alteration in mucosal permeability and deterioration in the function of the mucociliary escalator therefore increasing susceptibility to infection [2, 9]. Cigarette smoking also affects cell-mediated and humoral immune responses to infection, resulting in a number of seemingly contradictory influences on immune function, including both pro-inflammatory and immunosuppressive effects [10–13]. Such immunosuppressive effects may be expected to increase the severity and duration of infection [14–16]. Contrarily, there is some evidence that current smoking is associated with a decreased risk of mortality in pneumococcal pneumonia with bacteremia [17]. Given the complex nature of the effect of smoking on immune function, it is difficult to predict the overall impact of tobacco smoking on clinical outcomes in sepsis [5]. Furthermore, there is a paucity of published data and the results are conflicting with regard to the effects of smoking on sepsis-related morbidity and mortality [8, 18, 19]. Here, we describe the association between smoking status and patient outcomes in severe sepsis and septic shock.

## Methods

### Study population

The study was conducted at Lahey Hospital & Medical Center, a 317-bed tertiary care, academic hospital with approximately 40,000 emergency department visits annually. The institutional review board approved this study and waived the need for informed consent. Consecutive adults (≥ 18 years) discharged with a sepsis-related diagnosis between February 1, 2013, and January 30, 2014, were identified using ICD-9 codes (038, 0380, 0389, 77181, 78552, 99802, 99591, and 99592). The medical records were then manually reviewed to confirm that the patients met the criteria for either severe sepsis or septic shock. The sepsis-2 consensus definitions were utilized because data collection commenced prior to the publication of the sepsis-3 definition [20]. We included only patients meeting criteria for either severe sepsis or septic shock. In patients who had multiple episodes of severe sepsis or septic shock during a single hospitalization, only the first episode was included. Likewise, for patients with multiple admissions for severe sepsis or septic shock during the study period, solely their first admission was included.

Patients were excluded if they met the criteria for sepsis alone (without organ dysfunction), had systemic inflammatory response syndrome (SIRS) without a suspected infection, they had a history of immunosuppression (solid organ transplantation, stem cell transplantation; cytotoxic chemotherapy in the past 3 months; HIV infection; chronic corticosteroid therapy with ≥ 10 mg of prednisone or equivalent; neutropenia with an absolute neutrophil count < 1000/mm^3^; congenital immunodeficiency; or immunosuppressive therapy in the past 3 months, including azathioprine, methotrextate, TNF-alpha antagonists, or other biologic immunosuppressants), they were pregnant, smoking history was unavailable, were transitioned to comfort care only within 24 h of admission, and if treatment for sepsis was commenced at an outside hospital.

We retrospectively collected, from the medical record, demographics including age, gender, co-morbid conditions, sequential organ failure assessment (SOFA) score at the time of diagnosis with sepsis, and smoking status, as well as other predictors of outcomes related to sepsis. In order to characterize smoking status, the Center for Disease Control and Prevention’s (CDC) definitions of current, former, and never smoker were adapted [21]. A current smoker was defined as an individual who smoked ≥ 100 cigarettes and still smoked daily on admission or quit within 1 year of admission. A former smoker smoked ≥ 100 cigarettes and quit more than 1 year of admission. A never smoker never smoked a cigarette or smoked less than 100 cigarettes during her/his lifetime. A non-smoker was defined as the combination of all former and never smokers. In addition, data on sepsis-related outcomes, including intensive care unit (ICU) and hospital mortality, ICU and hospital length of stay, the need for and duration of treatment with vasopressors, the need for and duration of renal replacement therapy, and the need for and duration of mechanical ventilation were collected.

### Statistics

The primary outcome was inpatient mortality, and secondary outcomes were hospital length of stay, ICU length of stay, and the need for vasopressors, dialysis, and mechanical ventilation. The continuous variables were tested for normality. Nonparametric continuous variables were compared between current smokers, never smokers, and former smokers using Wilcoxon rank-sum test. Categorical variables were compared using chi-square test. The impact of smoking and other covariates on mortality was explored. Variables showing a significant difference (*p* ≤ 0.10) in univariate analysis were included in the multivariable logistic regression analysis. Chronic obstructive pulmonary disease (COPD) was also included in the multivariable analysis as the authors felt that clinically, it was an important mediator in the association. The statistical analysis for this study was generated using Statistical Analysis Software (SAS), version 9.4 for Windows.

## Results

### Patient characteristics

**Fig. 1:**
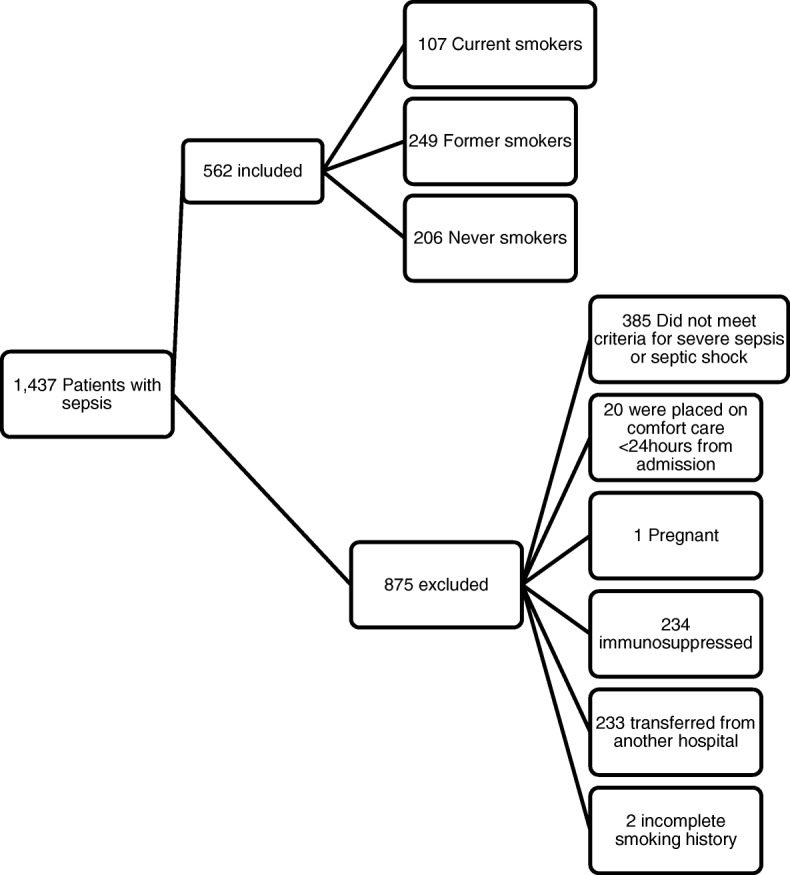
Patient enrollment. This is a study flow chart of patients with severe sepsis and septic shock based on smoking history

There were 1437 patients identified with a sepsis-related discharge diagnosis based on ICD-9 codes. After manually reviewing the medical records, 562 individuals met criteria for inclusion in this study (Fig. 1). Current smokers accounted for 19% (107/562) of patients, and 81% (455/562) were non-smokers. As a reference, the prevalence of smoking reported in the state of Massachusetts was 17% (95% CI, 16–18) for the year this study was conducted [22]. Of the non-smokers in our study, 55% (249/455) were former smokers and the rest (45%, 206/455) never smoked. Details on smoking history were available for 86/107(80%) of current smokers. The mean number of cigarettes smoked per day in this group was 23.3 ± 13.4, and the mean number of pack years smoked was 45.7 ± 30.5. In former smokers, a detailed smoking history was available in 176/249 (71%) of patients. In this group, the mean number of cigarettes smoked per day was 22.9 ± 13.5 and the mean number of pack-years smoked was 37.1 ± 25.6. The majority of patients were male (62%, 347/562), and there was no difference in gender distribution among the groups (Table 1). Current smokers were significantly younger than non-smokers (age 58, IQR 50–69 vs 78, IQR 66–85, *p* < 0.01). The median Charlson Comorbidity Index (CCI) was significantly lower in current smokers compared to non-smokers (4, IQR 2–5 vs 5, IQR 4–7, *p* < 0.01). Current smokers had fewer cardiac, neurologic, and renal co-morbidities but had more cirrhosis, COPD, and alcohol abuse than non-smokers (Table 1).

**Table 1:** Baseline characteristics stratified by smoking history

Demographics	All (*N* = 562)	Current smokers (*N* = 107)	Non-smokers (*N* = 455)	*P* value* (current vs. non)	Former smokers (*N* = 249)	*P* value** (current vs. former)	Never smokers (*N* = 206)	*P* value^§^ (current vs. never)
Age—median years (IQR)	74 (61–84)	58 (50–69)	78 (66–85)	< 0.0001	78 (69–85)	< 0.0001	76.5 (61–85)	< 0.0001
Male—*n* (%)	347 (62%)	69 (65)	278 (61)	0.52	166 (67)	0.69	112 (54)	0.09
BMI, median (IQR)	26 (23–31)	26 (23–32)	26 (23–31)	0.87	26 (23–31)	0.66	26 (22–31)	0.86
FEV1 mean (std)^∫^	1.76 (0.78)	2.11 (1.05)	1.65 (0.64)	0.048	1.64 (0.64)	0.049	1.67 (0.65)	0.09
FEV1 % pred mean (std)^∫^	68.4 (24.5)	66.6 (23.3)	68.9 (24.9)	0.69	69.3 (24.2)	0.65	67.9 (27.5)	0.87
FVC mean (std) ^∫^	2.54 (0.97)	3.07 (1.13)	2.37 (0.85)	< 0.01	2.39 (0.83)	0.004	2.32 (0.93)	0.03
FVC % pred mean (std)^∫^	73.2 (20.9)	75.9 (20.5)	72.3 (21.0)	0.46	73.0 (19.0)	0.54	70.2 (26.6)	0.44
FEV1/FVC mean (std)^∫^	68.6 (15.9)	67.3 (16.0)	69.0 (15.9)	0.65	69.1 (14.6)	0.64	68.7 (19.8)	0.79
Hospital acquired sepsis^#^	52 (9.3%)	15 (14%)	37 (8.1%)	0.06	21 (8.4%)	0.11	16 (7.8%)	0.08
Charlson Score, median (IQR)	5 (4–7)	4 (2–5)	5 (4–7)	< 0.0001	6 (4–7)	< 0.0001	5 (3–7)	0.0005
MI, *n* (%)	151 (27)	19 (18)	132 (29)	0.002	93 (37)	0.0003	39 (19)	0.80
CHF, *n* (%)	137 (24)	14 (13)	123 (27)	0.003	75 (30)	0.0007	48 (23)	0.03
CVA, *n* (%)	75 (13)	6 (6)	69 (15)	< 0.01	38 (15)	0.01	31 (15)	0.01
Dementia, *n* (%)	64 (11)	3 (3)	61 (13)	< 0.01	34 (14)	0.002	27 (13)	0.003
COPD, *n* (%)	110 (20)	35 (33)	75 (17)	< 0.01	68 (27)	0.30	7 (3)	< 0.0001
DM, *n* (%)	161 (29)	27 (25)	134 (30)	0.39	72 (29)	0.48	62 (30)	0.37
Cirrhosis, *n* (%)	56 (10)	24 (22)	32 (7)	< 0.01	15 (6)	< 0.0001	17 (8)	0.0004
CKD, *n* (%)	55 (10)	5 (5)	50 (11)	0.05	33 (13)		17 (8)	
Source of infection								
Pneumonia, *n* (%)	241 (43)	53 (50)	188 (41)	0.12	116 (47)	0.61	72 (35)	0.01
Genitourinary	154 (27)	21 (20)	133 (29)	0.05	66 (27)	0.17	67 (32)	0.02
Abdominal	108 (19)	31 (29)	77 (17)	< 0.01	42 (17)	0.01	35 (17)	0.01
Soft tissue	39 (7)	2 (2)	37 (8)	0.02	23 (9)	0.01	14 (7)	0.06
CNS	3 (1)	1 (1)	2 (0.4)	0.53	0 (0)	0.13	2 (1)	0.98
Cardiovascular	9 (2)	2 (2)	7 (2)	0.81	2 (1)	0.38	5 (2)	0.75
Blood stream	73 (13)	12 (11)	61 (13)	0.54	28 (11)	0.99	33 (16)	0.25
CVC	10 (2)	0 (0)	10 (2)	0.12	4 (2)	0.19	6 (3)	0.07
Other	0	0	0	–	0	–	0	–
Unknown	16 (3)	2 (2)	14 (3)	0.50	6 (2)	0.75	8 (4)	0.34
Medical, *n* (%)	481 (86)	84 (79)	397 (87)	0.02	218 (88)	0.03	179 (87)	0.05
Surgical	81 (13)	23 (22)	58 (13)		31 (13)		27 (13)	
Alcohol abuse, *n* (%)	64 (11)	37 (35)	27 (6)	< 0.01	19 (8)	< 0.0001	8 (4)	< 0.0001
Severe sepsis, *n* (%)	319 (57)	47 (44)	272 (60)	< 0.01	144 (58)	0.02	128 (62)	0.002
Septic shock, *n* (%)	243 (43)	60 (56)	183 (40)		105 (42)		78 (38)	
Lactate—median (IQR)	2.1 (1.3–3.4)	2.1 (1.5–3.1)	2.05 (1.2–3.5)	0.53	2.0 (1.2–3.2)	0.53	2.1 (1.2–3.7)	0.61
SOFA score median (IQR)	6 (3–9)	7 (4–12)	5 (3–9)	< 0.01	5 (3–9)	0.001	5 (3–8)	< 0.0001
Respiratory^‡^ median (IQR)	1 (0–2)	2 (0–3)	1 (0–2)	< 0.001	1 (0–3)	0.015	0 (0–2)	< 0.0001
72 h fluid balance—median L (IQR)	2.1 (0.2–4.35)	2.3 (0.01–5.9)	2.1 (0.26–4.1)	0.32	2.1 (0.01–4.1)	0.18	2.2 (0.5–4.1)	0.67
Stress steroids given—*n* (%)	38 (7)	9 (8)	29 (6)	0.45	17 (7)	0.60	12 (6)	0.38
Time to appropriate antibiotics after hypotension—minutes, median (IQR)	60 (− 60–159)	35 (− 117–120)	61 (− 60–165)	0.23	60 (− 76–165)	0.44	68.5 (− 22–170)	0.14
Appropriate empiric antibiotics given based on culture data								
Yes, *n* (%)	248 (44)	53(50)	195/454 (43)	0.17	107 (43)	0.17	88/205 (43)	0.26
No, *n* (%)	50 (9)	5(5)	45/454 (10)		26 (10)		19/205 (9)	
Unknown, *n* (%)	263 (47)	49(46)	214/454 (47)		116 (47)		98/205 (48)	

Abbreviations: *IQR* interquartile range, *BMI* body mass index, *std.* standard deviation, *FEV1* forced expiratory volume in 1 s, *FVC* forced vital capacity, *MI* myocardial infarction, *CHF* congestive heart failure, *CVA* cerebrovascular accident, *COPD* chronic obstructive pulmonary disease, *DM* diabetes mellitus, *CKD* chronic kidney disease, *CNS* central nervous system, *CVC* central venous catheter, *SOFA* sequential organ failure assessment

*P value refers to comparison between current smokers and non-smokers

**P value refers to comparison between current smokers and former smokers

^§^*P* value refers to comparison between current smokers and never smokers

^∫^There were only 98/562 (17.4%) patients that had available pulmonary function testing recorded prior to data collection. Therefore, the *N* in this section is unique: current smokers *N* = 24, former smoker *N* = 55, never smoker *N* = 19. Additionally, the reported spirometry is pre-bronchodilator

^#^Individuals who acquired sepsis more than 48 h following hospital admission

^‡^Independent evaluation of respiration as part of SOFA score. 0 = PaO_2_/FiO_2_ ratio > 400, 1 = PaO_2_/FiO_2_ 301–400 or SaO2/FiO_2_ 221–301, 2 = PaO_2_/FiO_2_ 201–300 or SaO2/FiO_2_ 142–220, 3 = PaO_2_/FiO_2_ 101–200 or SaO2/FiO_2_ 67–141, 4 = PaO_2_/FiO_2_ < 100 or SaO2/FiO_2_ 67

The overall percentage of patients with severe sepsis was 57% (319/562) and 43% (243/562) had septic shock. The proportion of patients with septic shock was significantly higher among current smokers (56%, 60/107 vs 40%, 183/455; *p* < 0.01), and pneumonia was the commonest source of sepsis among all groups. Abdominal sepsis was commoner in current smokers, whereas soft tissue and genitourinary infections were more frequently seen in non-smokers (Table 1). Current smokers had a significantly higher SOFA score than non-smokers at the time of diagnosis of severe sepsis or septic shock (7, IQR 4–12 vs 5, IQR 3–9; *p* < 0.01). Specifically, the respiratory component of the SOFA score was higher in the current smokers versus non-smokers (2, IQR 0–3 vs 1, IQR 0–2; *p* < 0.001).

### Clinical outcomes

The proportion of patients who died during their hospital stay was higher in current smokers (32%, 34/107) when compared to non-smokers (22%, 101/455, *p* = 0.04) (Table 2). In the multivariable analysis, when current smokers were compared to non-smokers, there was a trend towards higher mortality among current smokers but this failed to meet statistical significance (OR 1.81, 95% CI 0.92–3.54, *p* = 0.08) (Table 3). In comparison with former smokers, mortality was higher among current smokers (OR 2.18, 95% CI 1.002–4.743, *p* < 0.05) (Table 4). This association was not observed when mortality was compared between current smokers and never smokers (OR 1.47, 95% CI 0.612–3.516, *p* = 0.39) (Table 5). The percentage of patients who required ICU admission was higher in current smokers versus non-smokers (70% 75/107 vs 53% 243/455, *p* < 0.01). There was a trend towards a longer ICU length of stay in survivors in that same cohort (6, IQR 3–11 vs 4, IQR 2–10 days, *p* = 0.06). The overall hospital length of stay in survivors was significantly longer when comparing current smokers versus non-smokers (8, IQR 4–18 vs 7 IQR 4–12 days, *p* = 0.03). There was a greater need for mechanical ventilation (58 vs 29%, *p* < 0.01) and vasopressors (53 vs 39%, *p* < 0.01) in current smokers vs non-smokers, respectively, though the ventilator days, vasopressor days, the need for dialysis, and dialysis days did not significantly differ between the compared groups (Table 2). After controlling for other confounders, current smoking predicted the need for mechanical ventilation (OR 2.38, 95% CI 1.06–5.34, *p* = 0.04) but did not predict the need for vasopressors or ICU admission (Tables 6, 7, and 8).

**Table 2:** Outcomes in smokers, former smokers, and never smokers with severe sepsis or septic shock

Outcomes	Current smokers (*N* = 107)	Non-smokers (*N* = 455)	*P* value*	Former smokers (*N* = 249)	*P* value^**^	Never smokers (*N* = 206)	*P* value^§^
Mortality^‡^—*n* (%)	34 (32)	101 (22)	0.04	60 (24)	0.13	41 (20)	0.02
Hospital LOS—median days (IQR)	7 (4–17)	7 (4–12)	0.19	7 (4–12)	0.26	7 (4–12)	0.20
Hospital LOS in survivors	8 (4–18) (*N* = 73)	7 (4–12) (*N* = 354)	0.03	7 (4–12) (*N* = 189)	0.05	7 (4–11) (*N* = 165)	0.05
ICU LOS	5 (3–10) (*N* = 75)	4 (2–11) (*N* = 243)	0.36	5 (2–11) (*N* = 135)	0.67	4 (2–10) (*N* = 108)	0.20
ICU LOS survivors	6 (3–11) (*N* = 47)	4 (2–10) (*N* = 162)	0.06	4 (2–10) (*N* = 84)	0.10	4 (2–10) (*N* = 78)	0.07
Required ICU admission—*n* (%)	75 (70)	243 (53)	< 0.01	135 (54)	< 0.01	108 (52)	< 0.01
Required vasopressors—*n* (%)	57 (53)	175 (39)	< 0.01	104 (42)	0.05	71 (34)	< 0.01
Vasopressor days—median (IQR)	3 (2–4) (*N* = 57)	3 (2–5) (*N* = 175)	0.97	3 (2–5.5) (*N* = 104)	0.72	3 (2–5) (*N* = 71)	0.68
Required mechanical ventilation—*n* (%)	62 (58)	133 (29)	< 0.01	77 (31)	< 0.01	56 (27)	< 0.01
Ventilator days	5 (2–9) (*N* = 62)	5 (2–9) (*N* = 133)	0.99	6 (2–11) (*N* = 77)	0.42	4 (2–8) (*N* = 56)	0.32
Required dialysis—*n* (%)	10 (9)	42 (9)	0.97	27 (11)	0.67	15 (7)	0.52
Dialysis days	4 (2–6) (*N* = 10)	4 (2–9) (*N* = 42)	0.58	6 (3–13) (*N* = 27)	0.30	3 (2–6) (*N* = 15)	0.74

*LOS* length of stay, *ICU* intensive care unit

*P value refers to comparison between current smokers and non-smokers

**P value refers to comparison between current smokers and former smokers

^§^*P* value refers to comparison between current smokers and never smokers

^‡^Includes death or discharge to hospice

**Table 3:** Multivariable logistic regression analysis of active smoking and mortality in severe sepsis and septic shock (current smokers vs. non-smokers)

Risk factor	Odds ratio (95% CI)	*P* value
Current smoking	1.81 (0.92–3.54)	0.08
Alcohol abuse	1.17 (0.54–2.49)	0.69
72 h fluid balance	1.06 (0.99–1.13)	0.08
Age	1.02 (0.99–1.05)	0.06
Charlson score	1.16 (1.02–1.31)	0.02
SOFA	1.15 (1.068–1.238)	< 0.01
COPD	0.98 (0.539–1.794)	0.96
Hospital acquired sepsis	1.51 (0.711–3.201)	0.28
Severe sepsis	0.54 (0.288–1.011)	0.05
Genitourinary infect	0.52 (0.282–0.970)	0.04
Abdominal	0.93 (0.487–1.757)	0.81
Soft tissue	1.03 (0.394–2.713)	0.95
Medical ICU admission	1.62 (0.765–3.411)	0.21

All survival predictors (except for “respiratory”) that were noted to be significant (*p* < 0.1) on univariate analysis were included in this multivariate model

*SOFA* sequential organ failure assessment, *COPD* chronic obstructive pulmonary disease

**Table 4:** Multivariable logistic regression analysis of active smoking and mortality in severe sepsis and septic shock (current smokers vs. former smokers)

Risk factor	Odds ratio (95% CI)	*P* value
Current smoking	2.18 (1.00–4.74)	0.049
Alcohol abuse	1.61 (0.67–3.85)	0.29
72 h fluid balance	1.03 (0.95–1.12)	0.42
Age	1.02 (0.99–1.05)	0.17
Charlson score	1.18 (1.00–1.38)	0.05
SOFA	1.15 (1.05–1.25)	< 0.01
COPD	0.98 (0.49–1.93)	0.94
Hospital acquired sepsis	1.77 (0.704–0.4.442)	0.23
Severe sepsis	0.40 (0.176–0.895)	0.03
Genitourinary infect	0.29 (0.12–0.71)	< 0.01
Abdominal	0.95 (0.43–2.10)	0.90
Soft tissue	1.99 (0.61–6.51)	0.25
Medical ICU admission	1.65 (0.66–4.15)	0.29

**Table 5:** Multivariable logistic regression analysis of active smoking and mortality in severe sepsis and septic shock (current smokers vs. never smokers)

Risk factor	Odds ratio (95% CI)	*P* value
Current smoking	1.47 (0.61–3.52)	0.39
Alcohol abuse	0.73 (0.29–1.86)	0.51
72 h fluid balance	1.04 (0.95–1.13)	0.45
Age	1.03 (0.99–1.06)	0.10
Charlson score	1.21 (1.02–1.43)	0.03
SOFA	1.12 (1.02–1.24)	0.02
COPD	1.62 (0.59–4.41)	0.35
Hospital acquired sepsis	1.13 (0.40–3.17)	0.28
Severe sepsis	0.65 (0.29–1.01)	0.82
Genitourinary infect	0.86 (0.39–1.90)	0.71
Abdominal	0.98 (0.42–2.33)	0.97
Soft tissue	0.557 (0.10–3.08)	0.50
Medical ICU admission	1.06 (0.39–2.84)	0.91

**Table 6:** Multivariable analysis of active smoking and requiring ICU admission in severe sepsis and septic shock (current smokers vs. non-smokers)

Risk factor	Odds ratio (95% CI)	*P* value
Current smoking	0.93 (0.41–2.13)	0.86
Alcohol abuse	0.83 (0.31–2.23)	0.71
72 h fluid balance	1.04 (0.94–1.16)	0.42
Age	0.987 (0.96–1.01)	0.31
Charlson score	1.03 (0.89–1.19)	0.70
SOFA	1.42 (1.27–1.60)	< 0.01
COPD	1.35 (0.67–2.76)	0.40
Hospital acquired sepsis	3.87 (1.11–13.5)	0.03
Severe sepsis	0.08 (0.04–0.16)	< 0.01
Genitourinary infect	0.55 (0.29–1.04)	0.07
Abdominal	1.02 (0.44–2.35)	0.97
Soft tissue	0.95 (0.27–3.26)	0.93
Medical ICU admission	0.71 (0.27–1.91)	0.50

**Table 7:** Multivariable analysis of active smoking and requiring vasopressors in severe sepsis and septic shock (current smokers vs. non-smokers)

Risk factor	Odds ratio (95% CI)	*P* value
Current smoking	2.10 (1.01–4.36)	0.58
Alcohol abuse	1.25 (0.50–3.10)	0.99
72 h fluid balance	1.05 (0.98–1.13)	< 0.01
Age	1.02 (0.98–1.07)	0.34
Charlson score	0.89 (0.67–1.176)	0.40
SOFA	1.59 (1.34–1.88)	< 0.01
COPD	1.62 (0.39–6.68)	0.51
Hospital acquired sepsis	3.94 (0.61–25.4)	0.15
Severe sepsis	0.004 (0.001–0.01)	< 0.01
Genitourinary infect	1.01 (0.33–3.03)	0.99
Abdominal	0.61 (0.17–2.19)	0.45
Soft tissue	1.03 (0.14–7.37)	0.98
Medical ICU admission	1.23 (0.28–5.43)	0.79

**Table 8:** Multivariable analysis of active smoking and requiring mechanical ventilation in severe sepsis and septic shock (current smokers vs. non-smokers)

Risk factor	Odds ratio (95% CI)	*P* value
Current smoking	2.38 (1.06–5.34)	0.04
Alcohol abuse	0.99 (0.37–2.66)	0.99
72 h fluid balance	1.05 (0.97–1.11)	0.21
Age	0.98 (0.96–1.01)	0.21
Charlson score	0.92 (0.78–1.09)	0.33
SOFA	1.53 (1.37–1.70)	< 0.01
COPD	1.13 (0.52–2.43)	0.76
Hospital acquired sepsis	1.25 (0.45–3.45)	0.66
Severe sepsis	0.45 (0.23–0.88)	0.02
Genitourinary infect	0.26 (0.12–0.55)	< 0.01
Abdominal	0.40 (0.18–0.88)	0.02
Soft tissue	0.31 (0.10–0.96)	0.04
Medical ICU admission	0.13 (0.05–0.33)	< 0.01

### Clinical outcomes in patients with pneumonia

There was no statistically significant difference in the proportion of patients with pneumonia who died during their hospital stay when current smokers (34%, 18/53) were compared to non-smokers (24%, 45/188, *p* = 0.14) and former smokers (28%, 33/116, *p* = 0.47) (Table 9). However, the percentage of deaths was significantly less in never smokers (12%, 12/72) when compared to current smokers (*p* = 0.03).

**Table 9:** Outcomes in smokers, former smokers, and never smokers with severe sepsis or septic shock due to pneumonia (*N* = 241)

Outcomes	Current smokers (*N* = 53)	Non-smokers (*N* = 188)	*P* value*	Former smokers (*N* = 116)	*P* value^**^	Never smokers (*N* = 72)	*P* value^§^
Mortality^‡^—*n* (%)	18 (34)	45 (24)	0.14	33 (28)	0.47	12 (17)	0.03
Hospital LOS—median days (IQR)	8 (5–20)	7 (4–12)	0.13	7.5 (4–13)	0.16	7 (5–12)	0.20
Hospital LOS in survivors	10 (5–21)	7 (4–12)	0.02	7 (4–13)	0.03	7 (5–12)	0.05
ICU LOS	7 (3–13)	6 (3–12)	0.70	6 (3–11)	0.77	6 (2–12)	0.68
ICU LOS survivors	8 (4–13)	5 (2–12)	0.14	4 (2–11)	0.14	6 (3–12)	0.28
Required ICU admission—*n* (%)	41 (77)	104 (55)	< 0.01	66 (57)	0.01	38 (53)	< 0.01
Required vasopressors—*n* (%)	33 (62)	72 (38)	< 0.01	48 (41)	0.01	24 (33)	< 0.01
Vasopressor days—median (IQR)	3 (2–5)	3 (2–6)	0.73	3 (2–6)	0.62	3 (2–5)	0.97
Required mechanical ventilation—*n* (%)	39 (74)	66 (35)	< 0.01	42 (36)	< 0.01	24 (33)	< 0.01
Ventilator days	5 (3–12)	6 (3–10)	0.78	6 (3–11)	0.60	4 (3–8)	0.85
Required dialysis—*n* (%)	6 (11)	15 (8)	0.45	10 (9)	0.58	5 (7)	0.39
Dialysis days	4.5 (3–10)	6 (2–14)	0.70	10 (4–14)	0.32	2 (2–2)	0.52

*LOS* length of stay, *ICU* intensive care unit

*P value refers to comparison between current smokers and non-smokers

**P value refers to comparison between current smokers and former smokers

^§^*P* value refers to comparison between current smokers and never smokers

^‡^Includes death or discharge to hospice

The percentage of patients who required ICU admission was higher in current smokers versus non-smokers (77% 41/53 vs 55% 104/188, *p* < 0.01). There was a longer overall hospital length of stay in survivors in that same cohort (10, IQR 5–21 vs 7, IQR 4–12 days, *p* = 0.02), but ICU length of stay did not differ. There was a greater need for mechanical ventilation (74 vs 35%, *p* < 0.01) and vasopressors (62 vs 38%, *p* < 0.01) in current smokers vs non-smokers, respectively. Ventilator days, vasopressor days, need for dialysis, and dialysis days did not significantly differ among the compared groups (Table 9).

## Discussion

Sepsis is one of the most expensive conditions treated in US hospitals, and its impact on the morbidity and mortality of those afflicted is substantial [23]. Tobacco smoking has well-documented effects on immune function, but the overall impact of smoking on clinical outcomes in sepsis has been poorly defined. Our results suggest that smoking is a potentially modifiable risk factor that can impact the course of those who have severe sepsis or septic shock. Despite being a younger group with fewer comorbidities, there was a trend towards a higher mortality in current smokers especially when compared to former smokers. Previous studies have primarily focused on the effect of smoking on outcomes in patients with respiratory infection, with conflicting results reported. For example, there is evidence that active smoking is associated with a higher mortality in patients with respiratory infections, particularly pneumonia and influenza [19, 24]. Contrarily, a large South African retrospective study noted that with the exception of tuberculosis, there was no significant risk of tobacco-attributable mortality from lung infections in smokers compared to non-smokers [25]. In other studies, bacteremic patients with pneumococcal pneumonia who were current smokers had decreased mortality or no difference in mortality when compared to non-smokers with the same infection [17, 26]. However, none of these studies looked at the effect of smoking on a broad population with sepsis [7, 18, 19]. To our knowledge, this is the first study to specifically examine the effect of active smoking on a general population of patients with severe sepsis and septic shock.

Our data also suggest that smokers have a higher proportion of sepsis-related organ dysfunction. We observed a greater percentage of septic shock and an increased need for vasopressors and mechanical ventilation in current smokers despite this population being younger and having fewer comorbidities. Furthermore, current smokers had a higher SOFA score at the time of diagnosis and were more likely to require ICU admission, suggesting a higher severity of illness on presentation and a more fulminant sepsis course. There is convincing biological plausibility for these findings considering the reported effects of smoking on several pro-inflammatory mediators, including TNFα, interleukin 6 and 8 NF-κB [27–30]. Furthermore, smoking increases levels of pro-inflammatory cytokines, including tumor necrosis factor (TNF) alpha and interleukin (IL)-6 [13]. Such an exaggerated pro-inflammatory response to microorganisms could potentially result in worse outcomes from sepsis. We also observed a longer hospital length of stay and a trend towards a longer ICU length of stay, suggesting a longer recovery from sepsis. This may be related to the immunosuppressive effects of smoking, which renders the host less able to combat infection. The increased rates of mechanical ventilation and worse hypoxia in current smokers can partially be explained by the higher rates of COPD in that group. Smokers also have an increased susceptibility to respiratory tract infections and acute respiratory distress syndrome (ARDS), which puts them at risk for respiratory failure and the need for mechanical ventilation [2–4].

Another possible explanation for the trend towards higher mortality in smokers is its association with worse health habits. In concordance with other studies, our data illustrate that smoking is accompanied by alcohol abuse [4, 31]. However, alcohol abuse was not an independent predictor of mortality in our multivariable analysis. In other studies, smoking was associated with less vaccination uptake [4, 32]. As a behavior pattern, smokers may potentially delay seeking medical attention and that is reflected in a higher severity of illness on presentation. This association has been observed in lung cancer patients, where smokers often avoided medical advice for lung cancer symptoms [33].

Because pneumonia was observed to be the most common source of infection and due to its association with smoking, we performed a subgroup analysis of patients with respiratory infections. Overall, the outcome trends were similar in patients with sepsis due to pneumonia as compared to outcomes observed in other sources of sepsis. However, as one would expect, the contrast between current smokers and non-smokers’ need for mechanical ventilation was more pronounced in patients with pneumonia.

Our study is subject to a number of limitations. This was a single-center study, thus limiting the generalizability of our findings. Furthermore, the retrospective study design and small sample size potentially introduce bias into the results. Because the data were collected retrospectively, the smoking history and spirometry were limited to what was documented in the medical record a priori. However, the proportion of smokers observed in our study (19%, CI 95% 16–22) is similar to the prevalence of smoking reported in the state of Massachusetts (17%, CI 95% 16–18) for the year this study was conducted [22]. A larger sample size detailing patients’ smoking history including pack years would have shed light on any potential dose-dependent effect on sepsis. Additionally, a prospective study design with the use of a validated biomarker to quantify tobacco exposure such as NNAL would have provided an objective measure to corroborate the smoking history. Finally, we did not determine whether the study patients received nicotine supplementation during their hospitalization and did not assess for second-hand smoke exposure in the non-smokers.

## Conclusions

This study identified that current smokers trended towards a higher mortality in severe sepsis and septic shock despite being younger and with fewer comorbid illnesses. In addition, current smoking was associated with more than a twofold increase in the need for mechanical ventilation. Thus, tobacco smoking may represent a modifiable risk factor for worse outcomes in severe sepsis and septic shock. This was an exploratory study to evaluate the effects of smoking on severe sepsis and septic shock. Ultimately, large-scale multicenter prospective case-control studies are needed to confirm our findings.

## Abbreviations

ARDS: Acute respiratory distress syndrome
BMI: Body mass index
CCI: Charlson Comorbidity Index
CDC: Center for Disease Control and Prevention
CHF: Congestive heart failure
CKD: Chronic kidney disease
CNS: Central nervous system
COPD: Chronic obstructive pulmonary disease
CVA: Cerebrovascular accident
CVC: Central venous catheter
DM: Diabetes mellitus
ICU: Intensive care unit
IL: Interleukin
IQR: Interquartile range
LOS: Length of stay
MI: Myocardial infarction
SIRS: Systemic inflammatory response syndrome
SOFA: Sequential organ failure assessment
TNF: Tumor necrosis factor
